# A Newly Emergent Turkey Arthritis Reovirus Shows Dominant Enteric Tropism and Induces Significantly Elevated Innate Antiviral and T Helper-1 Cytokine Responses

**DOI:** 10.1371/journal.pone.0144085

**Published:** 2015-12-11

**Authors:** Tamer A. Sharafeldin, Sunil K. Mor, Nader M. Sobhy, Zheng Xing, Kent M. Reed, Sagar M. Goyal, Robert E. Porter

**Affiliations:** 1 Department of Veterinary Population Medicine and Minnesota Veterinary Diagnostic Laboratory University of Minnesota, St. Paul, MN, 55108, United States of America; 2 Department of Veterinary and Biomedical Sciences, University of Minnesota, St. Paul, MN, 55108, United States of America; 3 Department of Pathology, Faculty of Veterinary Medicine, Zagazig University, Zagazig, 44519, Egypt; 4 School of Medicine, Nanjing University, Nanjing, 210093, China; CEA, FRANCE

## Abstract

Newly emergent turkey arthritis reoviruses (TARV) were isolated from tendons of lame 15-week-old tom turkeys that occasionally had ruptured leg tendons. Experimentally, these TARVs induced remarkable tenosynovitis in gastrocnemius tendons of turkey poults. The current study aimed to characterize the location and the extent of virus replication as well as the cytokine response induced by TARV during the first two weeks of infection. One-week-old male turkeys were inoculated orally with TARV (O’Neil strain). Copy numbers of viral genes were estimated in intestines, internal organs and tendons at ½, 1, 2, 3, 4, 7, 14 days Post inoculation (dpi). Cytokine profile was measured in intestines, spleen and leg tendons at 0, 4, 7 and 14 dpi. Viral copy number peaked in jejunum, cecum and bursa of Fabricius at 4 dpi. Copy numbers increased dramatically in leg tendons at 7 and 14 dpi while minimal copies were detected in internal organs and blood during the same period. Virus was detected in cloacal swabs at 1–2 dpi, and peaked at 14 dpi indicating enterotropism of the virus and its early shedding in feces. Elevation of IFN-α and IFN-β was observed in intestines at 7 dpi as well as a prominent T helper-1 response (IFN-γ) at 7 and 14 dpi. IFN-γ and IL-6 were elevated in gastrocnemius tendons at 14 dpi. Elevation of antiviral cytokines in intestines occurred at 7dpi when a significant decline of viral replication in intestines was observed. T helper-1 response in intestines and leg tendons was the dominant T-helper response. These results suggest the possible correlation between viral replication and cytokine response in early infection of TARV in turkeys. Our findings provide novel insights which help elucidate viral pathogenesis in turkey tendons infected with TARV.

## Introduction

Turkey reoviruses are associated with a variety of turkey enteric diseases [1, 2, 3, 4, 5, 6, 7, 8] and experimentally were found to replicate in intestines and bursa of Fabricius by 2–5 days post inoculation (dpi) [9]. These viruses induced atrophy of the bursa of Fabricius and lymphocytic depletion in both the spleen and bursa of Fabricius of experimentally infected 3-day-old specific pathogen free (SPF) and commercial turkey poults [10, 11].

Reoviruses were first isolated from ruptured tendons of turkeys with tenosynovitis/arthritis in 1980s [12, 13]. However, Koch’s postulates were not fulfilled because reovirus strains isolated from turkeys with tenosynovitis/arthritis did not induce tenosynovitis/arthritis when inoculated into footpads of 1-day-old poults [14]. Recently, newly emergent turkey reoviruses were isolated from gastrocnemius and digital flexor tendons of 15 to 18-week-old lame turkeys in the Midwestern USA and these viruses were shown to be genetically distinct from chicken reoviruses (CARV [15]). These newly emergent viruses, tentatively called turkey arthritis reoviruses (TARV), showed a unique ability, unlike turkey enteric reoviruses (TERV) and CARV, to induce histologic tenosynovitis by 4 weeks post challenge in 1-week-old turkey poults inoculated via the oral, intratracheal and footpad routes [16]. Specifically, TARV-O’Neil induced the highest histopathologic inflammation score in tendon sheath compared with other TARV strains and avian reoviruses as early as 1–2 weeks PI [16]. Surprisingly, in this work, no reovirus related lameness was observed in the infected turkeys up to 4 weeks PI. In another work [17], the previous results were confirmed when TARV-O’Neil via oral route induced histologic inflammation at 4 weeks of age (3 weeks PI) and lameness was first displayed at 8 weeks of age (7 weeks PI). From these two studies, the established experimental model considered histologic tenosynovitis as an acceptable early endpoint instead of late endpoint (Lameness) for the future experiments.

According to these results, the aim of the present study was to characterize the early pathogenesis and the resulting cytokine profile of TARV infection in turkey poults to understand reovirus- host interaction and the role of immune response in viral pathogenesis.

## Materials and Methods

### Poults

One-day-old male turkey poults (n = 80) were purchased from a commercial turkey hatchery. Birds were divided into two groups of 40 each and placed in air filtered isolators supplied with food and water *ad-libitum*. Five birds were bled upon arrival and serum was tested for reovirus antibodies using an avian reovirus ELISA kit (IDEXX, Westbrook, ME). In addition, fecal samples were collected from ten poults and tested for presence of reovirus by real-time reverse transcription-polymerase chain reaction (rRT-PCR) [18].

### Virus

The TARV-O’Neil strain isolated in 2011 from gastrocnemius/digital flexor tendons of lame turkeys in Minnesota, was obtained from Dr. Jack Rosenberger, AviServe, Newark, DE. The virus was propagated and titrated on QT-35 cells. Briefly, 300 μL of virus stock was inoculated on complete monolayer of 175cm^2^ flask. After adsorption for 1 hr at 37°C, MEM with 4% DHS (donor horse serum) and antibiotics was added and incubated at 37°C. Flask was observed daily to check for CPE (cytopathic effect). Flask was frozen after observing 80% CPE. After three freeze-thaw cycles, the infected cell culture suspension was centrifuged and supernatant was collected. Virus was then titrated on QT-35 cells to a titer of 10^5.5^ TCID_50_/ml. TARV-O’Neil was chosen because it induced the highest inflammation score in the tendons of turkeys experimentally infected via oral route compared with other strains of TARV [16].

### Experimental design

One group of poults (n = 40) was inoculated orally at 7 days of age with 0.2 ml of 10^5.5^ TCID_50_/ml of TARV-O’Neil and the second group (n = 40) was inoculated with 0.2 ml of virus-free MEM. The two groups of male poults were kept in two separate air filtered isolators and were supplied food ad libitum. Five birds from each group were euthanized by pentobarbital injection at 0, 1/2, 1, 2, 3, 4, 7 and 14 days post inoculation (dpi) and samples were collected as described below. The study was designed to be terminated at 2 weeks PI based on the finding that TARV-O’Neil induced significantly higher tenosynovitis histologic scores (Early end point) compared with other TARV strains and avian reoviruses as early as 1–2 weeks PI [16]. Procedures for housing, inoculation and euthanasia of birds were approved by the Institutional Animal Care and Use Committee (IACUC), University of Minnesota (Protocol No. 1205A14203)

### Histopathology

Samples from intestines (duodenum, jejunum and cecum), bursa of Fabricius, heart, liver, spleen, kidney, and intertarsal joint with gastrocnemius tendon were collected and fixed in 10% neutral-buffered formalin. Tissues were trimmed, processed, embedded in paraffin, sectioned at 5 microns, and stained with hematoxylin and eosin (H&E) for histologic examination.

### Viral gene copy numbers

Duodenum, jejunum, cecum, bursa of Fabricius, cloacal swab, heart, liver, spleen, kidney and gastrocnemius, and digital flexor tendons were collected and 100 mg of each tissue sample was homogenized in Hanks’ balanced salt solution (HBSS) containing 2% donor horse serum. The homogenate was then centrifuged at 1500 g for 20 min and the supernatant was subjected to RNA extraction using QIAamp Viral RNA mini kit (Qiagen, Valencia, CA). Swabs were treated as tissue samples. RNA was extracted using TRIZOL RNA extraction protocol (Life Technologies, Carlsbad, CA) from 200μl sample of whole blood (with anticoagulant). Copy numbers of the S4 gene were then determined by a previously developed quantitative RT-PCR method specific for turkey reovirus S4 gene [18]. It has been reported that one TCID_50_ of TARV-MN4 was equivalent to11.6± 0.2RNA copies of the S4 gene [18].

### Cytokine profile

Samples of intestines (duodenum, jejunum and cecum), spleen, and tendons at 0, 4, 7 and 14 dpi were tested for the presence of mRNA of eleven cytokines including proinflammatory cytokines [Interleukin 6 (IL-6), lipopolysaccharide-induced tumor necrosis-alpha factor (LITAF)]; antiviral cytokines [Interferon-α (IFN-α), IFN-β]; IL-2; T helper1 (Th1) (IFN-γ, IL-12); T helper 2 (Th2) (IL-4, IL-5); and T helper 17 (Th17) IL-17. The housekeeping gene [Glyceraldehyde 3-phosphate dehydrogenase (GAPDH)] was used to calibrate the reactions. GAPDH was reported to be a stable housekeeping gene in intestines of turkeys [19]. Segments of duodenum, jejunum, cecum, spleen, and leg tendons (gastrocnemius and digital flexor) were collected, immersed in RNA Later (Life Technologies) and kept frozen at -20 C until use. For RNA extraction, 100mg of tissue was homogenized with RLT lysis buffer in tubes with ceramic beads and allowed to settle for 10–15 min. The supernatant was subjected to total RNA extraction using RNeasy mini kit (Qiagen, Valencia, CA). RNA was subjected to reverse transcription using Primscript RT Master Mix (TAKARA BIO, Otsu, Shiga, Japan). Resulting DNA product was analyzed by PCR using SYBR^®^ Premix Ex Taq^™^ II (TAKARA BIO). Sequences of turkey cytokines were found in NCBI GenBank and primers were designed to amplify regions of these sequences (Table 1). The PCR reactions included the following stages; holding stage (50°C for 60 sec and 95°C for 30 sec), PCR cycling stage (95°C for 5 sec and 60°C for 30 sec) up to 40 cycles and melting curve stage (95°C for 15 sec, 60°C for 60 sec and 95°C for 15 sec). Relative expression levels were calculated using the 2 –^ΔΔC^_T_ method where ΔΔC_T_ = (ΔC_T_ target cytokine gene—ΔC_T_ Calibrator (GAPDH)) _Time_ x—(ΔC_T_ target cytokine gene—ΔC_T_ Calibrator (GAPDH)) _Time 0_ [20]. Analyses were performed in duplicate.

**Table 1:** List of cytokine genes and the primers used for RT-PCR amplification.

Cytokine	Accession number	Primers	Product size
GAPDH	GQ184819.1	F 5’CTGGCAAAGTCCAAGTGGTG3’	123 bp^a^
		R 5’TCCCATTCTCAGCCTTGACA3’	
IL-2	AJ007463.1	F 5’TGGAGCATCGCTATCACCAG3’	136 bp
		R 5’TTGCTGACTGCACTCCTTGA3’	
IL-4	XM_003210493.1	F 5’TTCCTGTGGCAAGATGAACG3’	124 bp
		R 5’CTGCAGGTTCTTGTGGCAGT3’	
IL-5	XM_003210491.1	F 5’TGACGAAAGCTGCATCAAAA3’	134 bp
		R 5’CTCTTGCCAGGTTTGCTGTG3’	
IL-6	XM_003207130.1	F 5’GCTTCGACGAGGAGAAATGC3’	120 bp
		R 5’AGCACAGCGATTCGACATTC3’	
IL-10	AM493432	F 5’TGGGCCTGAAGATGACAATG3’	131 bp
		R 5’CTCCCCCATGGCTTTGTAGA3’	
IL-12	AJ564203.1	F 5’TCCAAAGACTGGGCCAAAAG3’	121 bp
		R 5’CTCCAGCAGCAGAAGGCTCT3’	
IL-17	XM_003204633.1	F 5’CCATTGCTGTTGGTGTTGCT3’	115 bp
		R 5’GGCATCCAGCATCTCCTTTC3’	
IFN-α	U28140.1	F 5’GCCTCCTCAACCAGATCCAG3’	108 bp
		R 5’TGATGGTGAGGTGAGGGTTG3’	
IFN-β	XM_003213368.1	F 5’CCGTTCTGGAAAGCAAGGAC3’	119 bp
		R 5’GTGTGCGTGGTCAATCCAGT3’	
IFN-γ	AJ000725	F 5’ACCTGGCCAAGCTTCAGATG3’	115 bp
		R 5’TGGCTCCTTTTCCTTTTGGA3’	
LITAF	XM_003210543.1	F 5’TGACTTGGCTGTCGTGTGGT3’	119 bp
		R 5’GGCATTGCAATTTGGACAGA3’	

^a^ Base pairs

### Statistical analysis

Average Ct values were compared between infected and non-infected groups using parametric student’s *t*-test and statistical significant difference was considered at P < 0.05. The non-parametric Mann Whitney U test was used to test for significant differences in the median virus gene copy number between time points in different tissues.

## Results

### Histopathology

**Fig 1:**
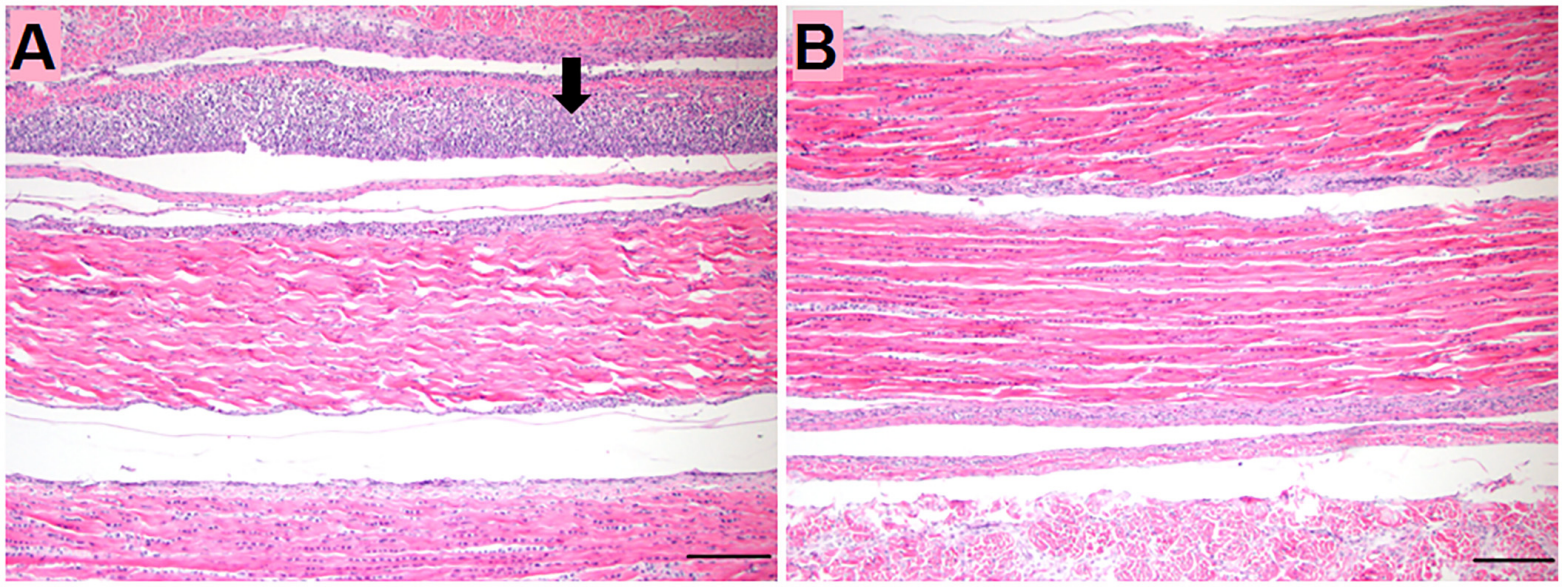
Histologic section in gastrocnemius tendon of turkeys at 14 dpi. (A) TARV-infected turkey at 14 dpi showing subsynovial lymphocytic infiltration (Arrow). (B) Non-infected control at 14 dpi. Bar = 100 μm.

No significant lesions were observed in sections of internal organs, intestines or intertarsal joint and tendons until 14dpi. However, gastrocnemius tendons showed tenosynovitis characterized by mild to moderate, diffuse subsynovial infiltration of lymphocytes (Fig 1).

### Virus gene copy numbers (Fig 2)

**Fig 2:**
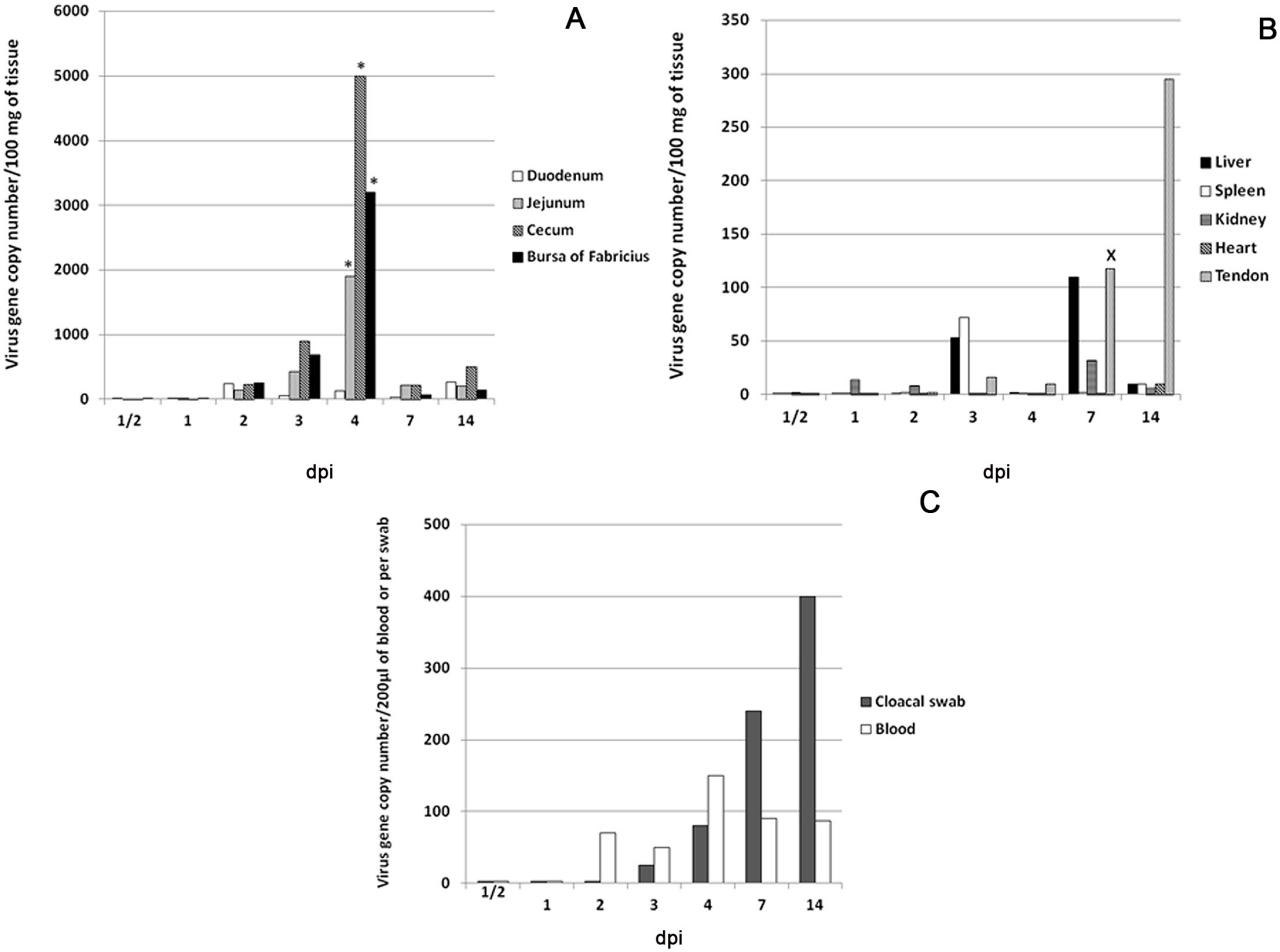
Viral gene copy numbers as determined by RT-PCR. Values represent the medians of five turkeys at each time point; (A) Intestines and bursa of Fabricius. Virus gene copy number significantly (P <0.05) peaked in jejunum, cecum and bursa of Fabricius at 4 dpi and a significantly declined at 7 dpi. (B) Internal organs have minimal gene copy number and a significant elevation (P <0.05) is present in tendons at 7 and 14 dpi. (C) Blood has minimal gene copy number that peaked at 4 dpi. Cloacal swabs, show detectable titer starting at 1–3 dpi and peaked at 14 dpi. Mann Whitney U test. *Significantly (P<0.05) higher than same tissue in other time points (before and after). ^X^ Significantly (P<0.05) higher than same tissue in the proceeding time points (before).

Copies of the S4 gene were detectable at 1–2 dpi in different intestinal segments and bursa of Fabricius. At 2 dpi, median viral gene copy numbers were (240, 140, 235 and 250)/100mg in duodenum, jejunum, cecum and bursa of Fabricius, respectively. Median gene copy numbers peaked at 4 dpi in jejunum, cecum and bursa of Fabricius (1800, 5000 and 3200) copies/100mg, respectively. These values were significantly higher (P<0.05) than values in the same intestinal segments at other time points before and after 4 dpi. At 7 dpi, gene copy numbers remarkably declined in all intestinal segments followed by slight elevation at 14 dpi, where the median peaked in the duodenum (260 copies/100mg) (Fig 2A).

In liver, kidney, spleen and heart, median gene copy numbers were under 100 copies/100mg at all-time points but were highest at 3 dpi in spleen and liver (53 and 72) copies/100mg and at 7 dpi in liver (110) copies/100mg. Copy number was low in tendon early but showed a dramatic and significant increase at 7 dpi (118 copies/100mg) (P<0.05). Viral load increased in tendon and at 14 dpi, reached 295 copies/100mg (Fig 2B).

In blood, median viral gene copy numbers peaked at 4 dpi (150 copies/200 μl). Viral load detected in the cloacal swabs increased at 3 and 4 dpi (25 and 80 copies/100 mg) and reached a peak at 14 dpi (400 copies/100mg) (Fig 2C)

### Cytokine profiling

#### Antiviral and anti-inflammatory cytokines

**Fig 3:**
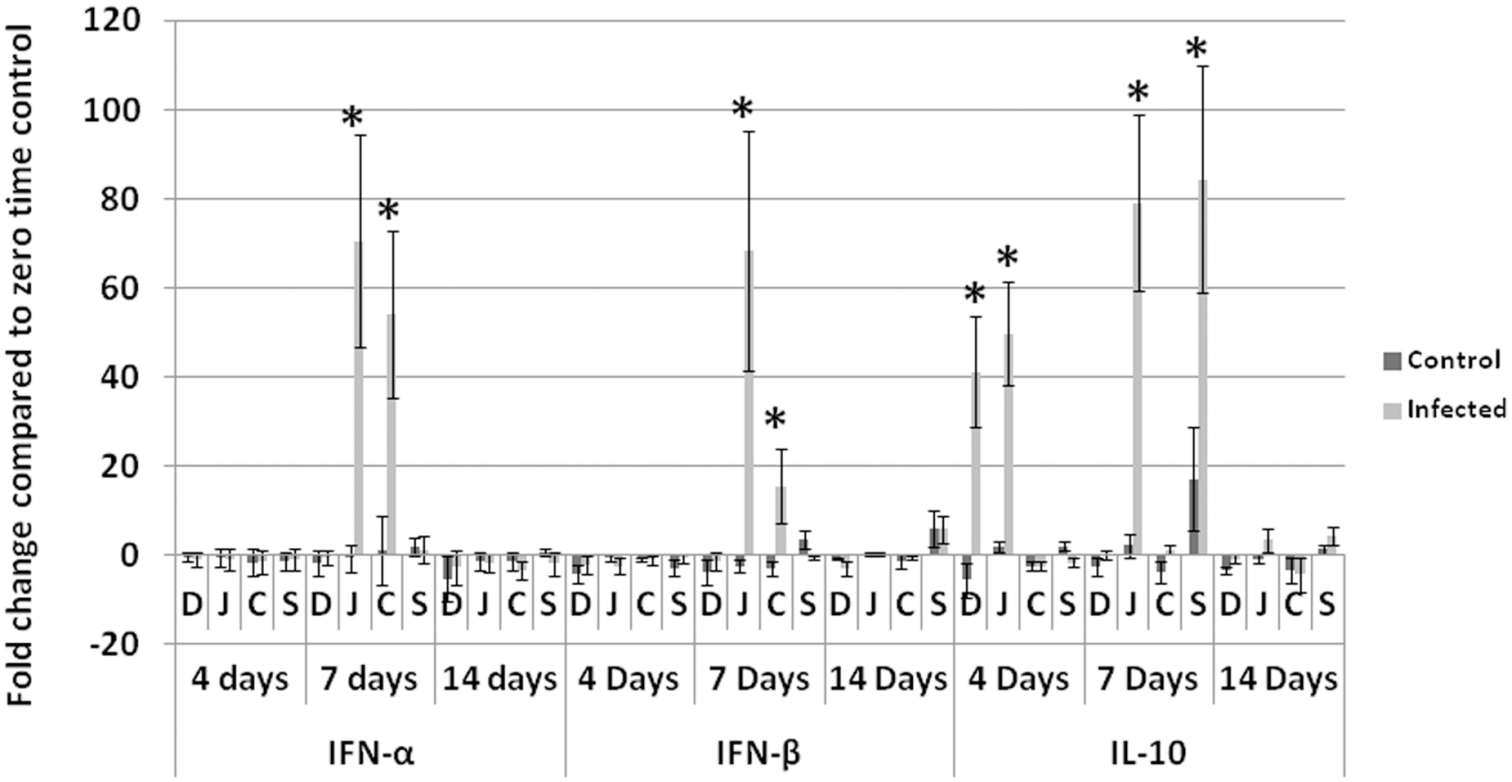
Fold change in antiviral cytokines (IFN-α and IFN-β) and anti-inflammatory IL-10. At 7 dpi IFN-α and IFN-β in jejunum and cecum of infected birds were significantly elevated. IL-10 is significatnly elevated in duodenum and jejunum of infected birds at 4 dpi and in jejunum and spleen of infected birds at 7 dpi. **D**: duodenum, **J**: jujenum, **C**: cecum, **S** spleen, **Days** refers to days post inoculation (dpi).* Analyses were performed in duplicate (Five turkeys/group at each time point) and values are mean ± 3SD. Differences between the infected and non-infected groups within the same organ are significant (P<0.05).

At 7 dpi, IFN-α and IFN-β were significantly higher in only the jejunum and cecum of infected groups compared with non-infected control. IL-10 showed significantly higher fold change in infected groups compared with non-infected control in duodenum and jejunum at 4 dpi and in jejunum and spleen at 7 dpi (Fig 3).

#### T helper 1, 2 and 17

**Fig 4:**
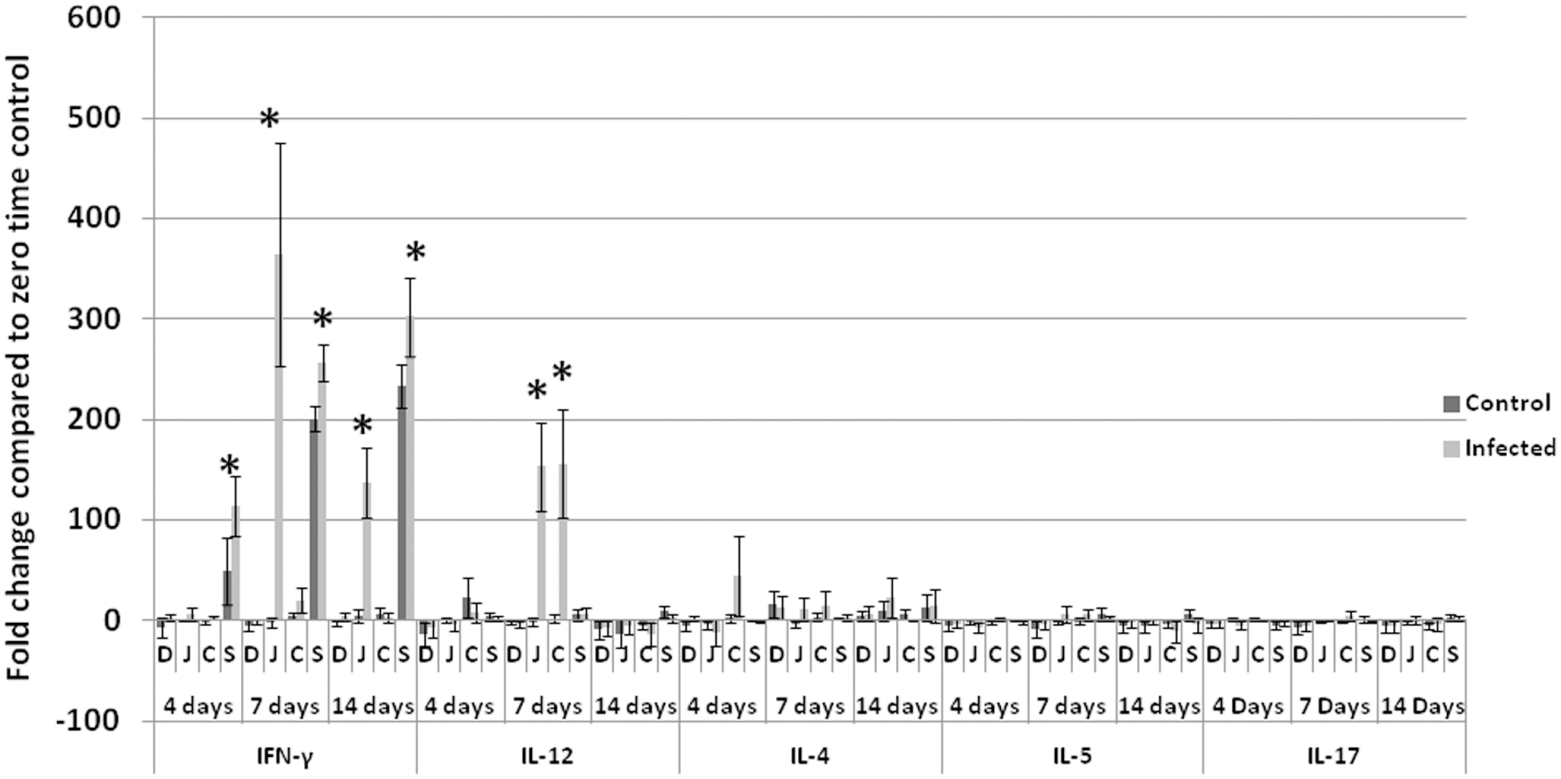
Fold changes in Th1, Th2 and Th17 cytokines observed in turkeys. Significant elevation of Th1 cytokine IFN-γ was observed in the jejunum of infected birds at 7 and 14 dpi and in spleen at 4, 7 and 14 dpi. The other Th1 cytokine, IL-12, shows significant elevation in jejunum and cecum of infected birds at 7 dpi. Th2 (IL-4 and IL-5) and Th17(IL-17) are not significantly different at P<0.05. **D**: duodenum, **J**: jujenum, **C**: cecum, **S** spleen, **Days** refers to days post inoculation (dpi). Analyses were performed in duplicate (Five turkeys/group at each time point) and values are mean ± 3SD. *Differences between infected group and non-infected groups within the same organ are significant (P<0.05).

**Fig 5:**
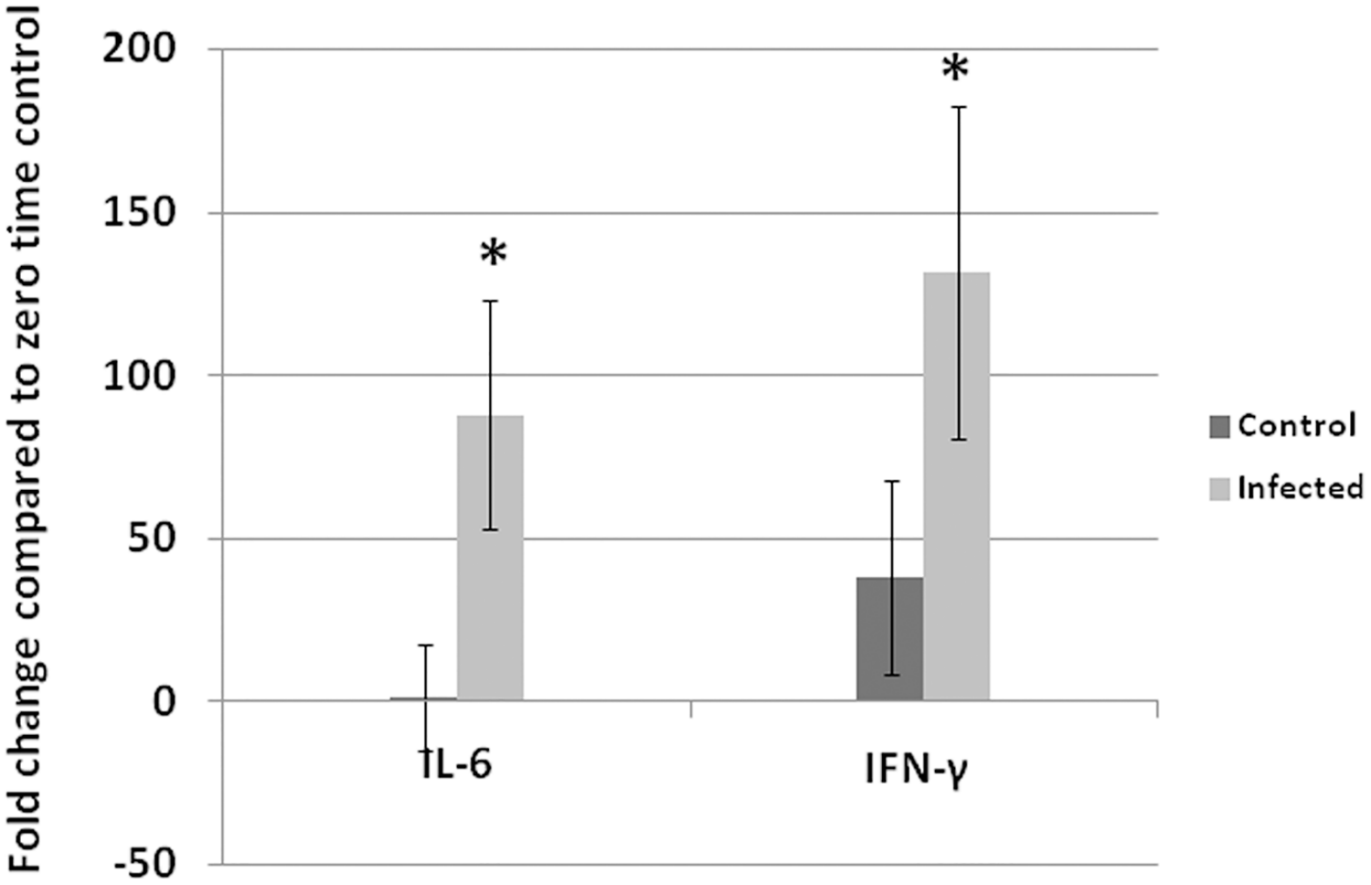
Fold change of IL-6 and IFN-γ in tendons at 14 dpi. Both IL-6 and IFN-γ were significantly elevated at 14 dpi in tendons of infected birds compared with non-infected control. Analyses were performed in duplicate (Five turkeys/group at each time point) and values are mean ± 3SD. *Differences between infected group and non-infected groups within the same organ are significant (P<0.05).

IL-12 showed significantly higher fold change in the infected group compared with non-infected control in jejunum and cecum at 7 dpi while IFN-γ increased significantly in jejunum at 7 and 14 dpi, spleen at 4, 7 and 14 dpi (Fig 4) and in tendons at 14 dpi (Fig 5). Th2 (IL-4 and IL-5) and Th17 (IL-17) cytokines did not show statistically significant differences between infected and non-infected control groups (Fig 4).

#### Proinflammatory cytokines

**Fig 6:**
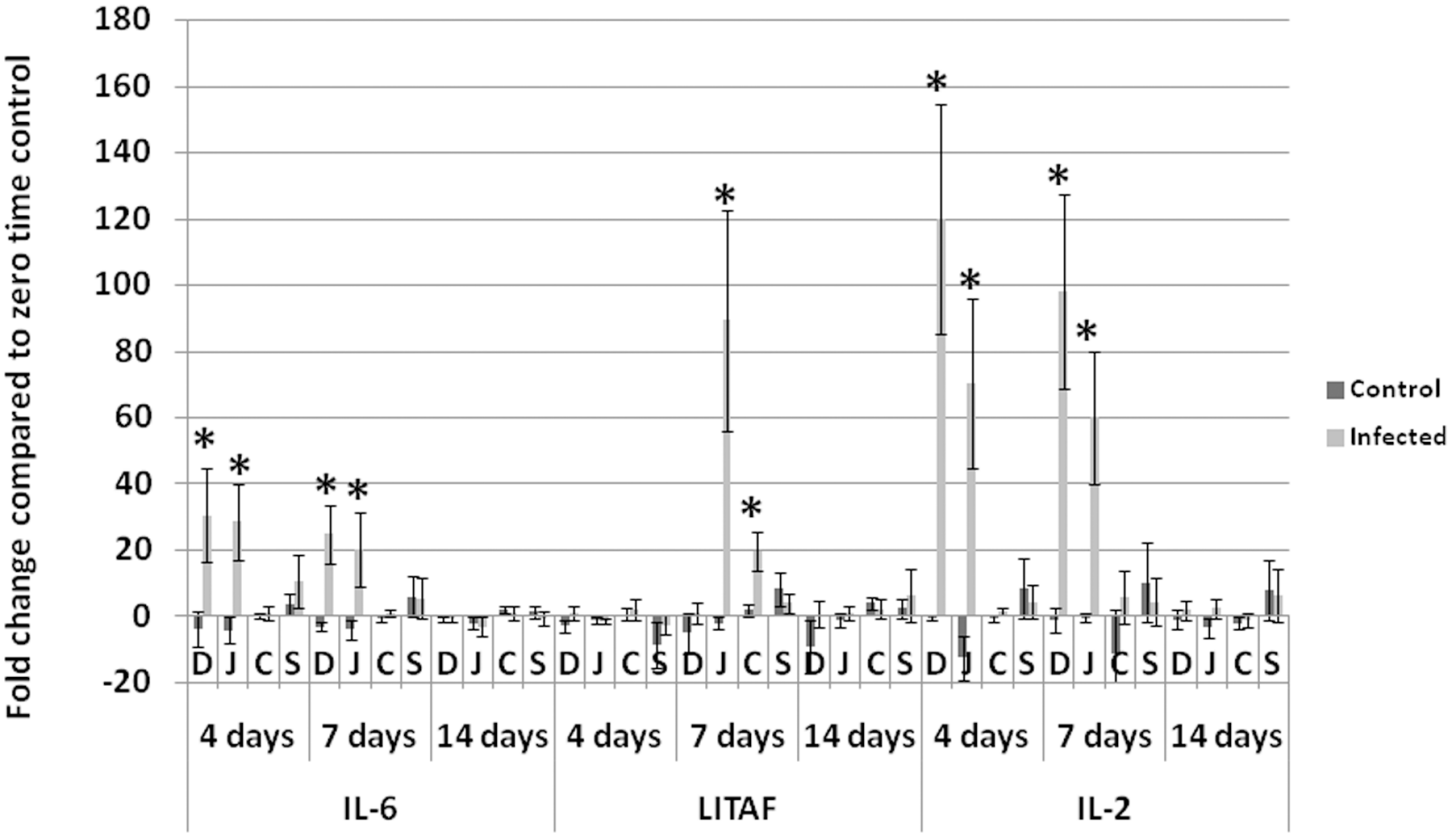
Fold change in proinflammatory cytokines (IL-6 and LITAF) and IL-2. IL-6 and IL-2 are significantly elevated in dudenum and jejunum of infected birds at 4 and 7 dpi. LITAF has significantly higher fold increase in infected birds compared with non-infected control in jejunum and cecum at 7 dpi. **D**: duodenum, **J**: jujenum, **C**: cecum, **S** spleen, **Days** refers to number of days post inoculation (dpi). Analyses were performed in duplicate (Five turkeys/group at each time point) and values are mean + 3SD. *Difference between infected group and non-infected groups within the same organ are significant (P<0.05).

Average fold changes of IL-6 were significantly higher in infected group than non-infected control in duodenum and jejunum at 4 and 7 dpi when compared with day zero (Fig 6). IL-6 was significantly elevated in tendons of infected birds at 14 dpi. Average fold changes of LITAF were significantly higher in infected groups only in jejunum and cecum at 7 dpi. IL-2 in duodenum and jejunum of infected birds had a significantly higher fold changes at 4 and 7 dpi.

## Discussion

The present work aimed to study tissue distribution and tropism of a newly emergent turkey reovirus associated with tenosynovitis/arthritis (TARV-O’Neil strain). Understanding tropism of this newly emergent virus helps in characterizing pathogenesis in tendons. Characterizing the cytokine profile induced by infection enhances our knowledge about the immune response in turkeys against the viral infection.

As demonstrated by rRT-PCR, the intestines (Jejunum and cecum) and bursa of Fabricius are the main sites of viral replication (based on the S4 gene copy number), peaking significantly (P<0.05) at 4 dpi. This technique can detect as few as 10 viral gene copies [18]. These findings are in agreement with the results of previous studies, which showed that the intestines and bursa of Fabricius were the initial sites of replication (within 2–5 dpi) of several TERVs [9] and chicken reoviruses [21]. Viral gene copy number was low in internal organs and blood compared with intestines and bursa of Fabricius while numbers increased significantly (P<0.05) in gastrocnemius tendon at 7 dpi, peaking at 14 dpi. Although the viral RNA was detected in blood at 2 dpi and peaked at 4 dpi, this low level of viremia was not associated with general systemic clinical illness at these time points. Chicken reoviruses have been shown to initially replicate in intestines, reach the blood at 2–3 dpi, and subsequently the internal organs within 3–5 dpi [22]. The low viral gene copies in blood in our study explains the absence of early systemic disease induced by TARV-O’Neil and failure of the virus to reach numbers in internal organs as high as in intestines and bursa of Fabricius. Further investigation of virus-host’s cells interaction will help understanding the reason of low viral load in blood and internal organs.

Reoviral pathogenesis has not been examined in turkey tendons. Previous studies of chickens inoculated with chicken reoviruses, targeted the hock joint, which was reported as an important site for virus replication [23, 24, 25]. In this report, we showed that the TARV-O’Neil strain replicated in tendons and reached peak copy number at 14 dpi. Only at this later time point was lymphocytic tenosynovitis observed in the gastrocnemius tendon sheath of infected birds. There were no lesions in tendons at earlier time points when viral gene copy number was low. These data indicate that inflammation in the tendon sheath was associated with the presence of a detectable virus titer.

Viral gene copy numbers measured in the cloacal swabs were indicative of early viral shedding starting at 1–2 dpi and peaking at 14 dpi. This may explain the rapid spread of infection among young birds in the field. In chicken, shedding of reovirus was reported at two weeks PI via oral route [26]. A separate study found shedding to peak at 1–2 weeks PI, and decrease after 3 weeks PI [27, 28]. TARV-O’Neil appears to have earlier shedding in turkeys, although this difference may be due to better early detection by the highly sensitive technique (rRT-PCR) [18].

Viral gene copies significantly (P<0.05) peaked at 4 dpi in intestines (jejunum and cecum) and bursa of Fabricius and then significantly (P<0.05) decreased at 7 dpi. This can be attributed to the antiviral effect of IFN-α and IFN-β, which were significantly elevated at 7dpi in jejunum and cecum of infected birds. This suggests that interferons played an important antiviral role in limiting TARV-O’Neil replication in intestines. We analyzed cytokine profile in multiple tissues of turkeys infected with the reovirus in order to understand the immunopathogenesis of the infection. In addition to the previous report of GAPDH stability in intestines of turkeys [19], we analyzed the mRNA expression (CT values) of GAPDH at different time points in each tissue using ANOVA and P values were more than 0.05. Insignificant difference confirmed the stability of GAPDH and its validity to be used as a house keeping gene. The significant elevation of IL-2 (P<0.05) in intestines of infected groups at 4 and 7 days, suggests proliferation of lymphocytes in response to viral infection. This proliferation likely caused infiltration of lymphocytes within the gut associated lymphoid tissues (GALT) such as Peyer’s patches, cecal tonsils or other intestinal lymphoid aggregates. Infiltration in the intestinal lamina propria was unlikely. Pro-inflammatory cytokines IL-6 and LITAF were significantly elevated in intestinal segments of infected birds; IL-6 in duodenum and jejunum of infected birds at 4 and 7 dpi and LITAF in jejunum at 7 dpi. Though IL-6 and LITAF significantly increased, they may not have been effective as there was no evidence of inflammation in the examined intestinal sections as represented by leukocyte infiltration, dilated blood vessels or exudation, usually associated with elevated proinflammatory cytokines at those time points. Similarly, IL-10 showed statistically significant elevation in intestines and spleen of infected birds at 4 and 7 dpi but apparently was not effective at down regulating Th1 cytokines in infected birds.

Th1 cytokines IFN-γ and IL-12 were significantly elevated in intestines and spleen of infected birds while Th-2 (IL-4 and IL-5) and Th-17 (IL-17) did not show significant elevation. This dominant Th1 response in intestines at 7 and 14 dpi excluded the possibility of an immunoglobulin role during the early course of infection and seemingly limits the possibility of an autoimmune reaction mediated by IL-17 [29]. The elevated IL-10 might be indicative of the activity of regulatory T (T reg) over Th17 which was supported by absence of destructive bone lesions in a long-term pathogenicity trial [17].

Comparing the cytokine response with viral replication and histologic alteration in leg tendons helps determine the events during the course of viral infection preceding lesions in leg tendons. Only at 14 dpi, did IL-6 and IFN-γ show significant elevations (P<0.05). This increase corresponds to subsynovial lymphocytic infiltration in gastrocnemius tendon sheath first observed at 14 dpi. It is likely this lymphocytic infiltration was induced by viral replication reaching a peak at 14 dpi.

In chickens, replication of reoviruses with high multiplication rate was shown to induce significantly higher production of IL-6, IL-10 and INF-γ compared to those with low multiplication rate [30]. Viral replication was accompanied with inflammation in leg tendons while in intestines, where replication was higher, inflammation was not a factor. Viral replication may be associated with inflammatory cells in GALT and lymphocytes infiltrating the tendon sheath. Little is known about the release of avian reoviruses and its association with cell lysis. Mammalian reoviruses may release from infected cells without inducing cell death [31] or may induce apoptosis prior to release [32]. We have not observed any syncytia formation by histologic examination, although avian reovirus is characterized by formation of cell-cell fusion (syncytia formation), mostly mediated by P10 protein [33]. Future studies using electron microscopy, specific immunohistochemistry and transcriptome analysis will be very helpful in determining details of the virus replication cycle including adhesion, assembly and release, as well as the cells where the virus replicates for local and systemic spread.

## Conclusions

The newly emergent turkey arthritis reovirus (TARV-O’Neil) is mostly enterotropic with a tendency to replicate in tendons later during the course of infection. The enterotropic virus is shed early during the course of infection in feces and evokes a significantly elevated antiviral cytokine response in intestines at 7 dpi when the viral replication was significantly decreased. Additionally, viral infection induced a dominant Th1 cytokine response but neither Th2 nor Th17 cytokines was elevated in infected birds during the first 2 weeks of infection. Further research is required to demonstrate viral pathogenesis at a later stage of infection when clinical lameness becomes evident.

## Supporting Information

S1 Table Means (M) and standard deviations (SD) of virus gene copy numbers at different days post inoculation in different organs (Per 100 mg of tissue or 200μl of blood or cloacal swab). (DOCX)

S2 Table Means and standard deviations of different cytokines fold changes in duodenum (D), jejunum (J), cecum (C), spleen (S) and tendon (T) in infected birds and non infected controls at different time points post inoculation (DOCX)
