# Supplementary material for: A Newly Emergent Turkey Arthritis Reovirus Shows Dominant Enteric Tropism and Induces Significantly Elevated Innate Antiviral and T Helper-1 Cytokine Responses
Source: PLoS One. 2015 Dec 11;10(12):e0144085. doi: 10.1371/journal.pone.0144085 (PMC4684236; doi:10.1371/journal.pone.0144085)
Supplement: S1 Table — (DOCX) [file pone.0144085.s001.docx]

| **Days post inoculation** | **Duodenum** | | **Jejunum** | | **Cecum** | | **Bursa of Fabricius** | | **Liver** | | **Spleen** | | **Kidney** | | **Heart** | | **Tendon** | | **Cloacal swab** | | **Blood** | |
| --- | --- | --- | --- | --- | --- | --- | --- | --- | --- | --- | --- | --- | --- | --- | --- | --- | --- | --- | --- | --- | --- | --- |
|  | **M** | **SD** | **M** | **SD** | **M** | **SD** | **M** | **SD** | **M** | **SD** | **M** | **SD** | **M** | **SD** | **M** | **SD** | **M** | **SD** | **M** | **SD** | **M** | **SD** |
| **1/2** | 0 | **0** | 0 | **0** | 0 | **0** | 0 | **0** | 0 | **0** | 0 | **0** | 0 | **0** | 0 | **0** | 0 | **0** | 0 | **0** | 0 | **0** |
| **1** | 0 | **0** | 22 | **19.2** | 10 | **8** | 17 | **14.41** | 0 | **0** | 0 | **0** | 14 | **8** | 0 | **0** | 0 | **0** | 0 | **0** | 0 | **0** |
| **2** | 200 | **100** | 110 | **80.94** | 204 | **198.07** | 220 | **160** | 0 | **0** | 0 | **0** | 8 | **8** | 0 | **0** | 0 | **0** | 0 | **0** | 60 | **54.75** |
| **3** | 48 | **40.69** | 450 | **250** | 822 | **539.19** | 634 | **438.26** | 44 | **38.78** | 60 | **58.87** | 0 | **0** | 0 | **0** | 16 | **18.88** | 14 | **10** | 40 | **22.53** |
| **4** | 114 | **96.17** | 1860 | **973.87** | 4438 | **1782.56** | 3038 | **1056.23** | 0 | **0** | 0 | **0** | 0 | **0** | 0 | **0** | 18 | **19.39** | 76 | **38.78** | 100 | **32.96** |
| **7** | 22 | **19.19** | 222 | **190.81** | 214 | **158.42** | 70 | **53.21** | 90 | **88.27** | 0 | **0** | 32 | **29.93** | 0 | **0** | 140 | **83.63** | 200 | **83.18** | 80 | **58.12** |
| **14** | 242 | **181.4** | 160 | **120** | 550 | **471.9** | 142 | **50.32** | 10 | **8.37** | 10 | **9.67** | 6 | **5.76** | 10 | **12.64** | 272 | **123.13** | 302 | **157.06** | 70 | **66.39** |

S1 Table: Means (M) and standard deviations (SD) of virus gene copy numbers at different days post inoculation in different organs (Per 100 mg of tissue or 200µl of blood or cloacal swab).
