# Supplementary material for: A Newly Emergent Turkey Arthritis Reovirus Shows Dominant Enteric Tropism and Induces Significantly Elevated Innate Antiviral and T Helper-1 Cytokine Responses
Source: PLoS One. 2015 Dec 11;10(12):e0144085. doi: 10.1371/journal.pone.0144085 (PMC4684236; doi:10.1371/journal.pone.0144085)
Supplement: S2 Table — (DOCX) [file pone.0144085.s002.docx]

| Cytokine  S2 Table: Means and standard deviations of different cytokines fold changes in duodenum (D), jejunum (J), cecum (C), spleen (S) and tendon (T) in infected birds and non infected controls at different time points post inoculation. | Days Post Inoculation | Organ | Control | | | | | Infected | | |
| --- | --- | --- | --- | --- | --- | --- | --- | --- | --- | --- |
|  |  |  | Mean | | | | SD | Mean | SD | |
| IFN-α | 4 days | D | -0.4487 | | | | 1.2354 | -0.734 | 1.675 | |
|  |  | J | -0.3349 | | | | 2.487 | -0.738 | 2.445 | |
|  |  | C | -1.5274 | | | | 3.668 | -1.31393 | 2.633 | |
|  |  | S | -1.2398 | | | | 2.8986 | -0.8319 | 2.415 | |
|  | 7 days | D | -1.739 | | | | 3.2987 | -0.40962 | 1.597 | |
|  |  | J | -0.5387 | | | | 3.654 | 70.69188 | 23.74 | |
|  |  | C | 1.221209 | | | | 7.944 | 54.11435 | 18.782 | |
|  |  | S | 2.1081 | | | | 2.764 | 1.313342 | 3.382 | |
|  | 14 days | D | -5.24987 | | | | 5.095 | -2.436 | 4.284 | |
|  |  | J | -1.2443 | | | | 2.2667 | -1.345 | 2.349 | |
|  |  | C | -1.138 | | | | 2.09895 | -3.6984 | 2.311 | |
|  |  | S | 0.8349 | | | | 1.45832 | -1.83459 | 2.8373 | |
| IFN-β | 4 Days | D | -4.06604 | | | | 2.02326 | -2.18613 | 2.4549 | |
|  |  | J | -0.35205 | | | | 0.769 | **-2.26694** | 2.392 | |
|  |  | C | -0.89776 | | | | 0.58645 | -0.9 | 1.4392 | |
|  |  | S | -2.8 | | | | 1.9024 | -0.56054 | 1.3819 | |
|  | 7 Days | D | -3.60776 | | | | 2.9254 | -1.15619 | 2.43856 | |
|  |  | J | -2.26957 | | | | 1.4637 | 68.4766 | 27.149 | |
|  |  | C | -2.9 | | | | 1.8679 | 15.66981 | 8.4249 | |
|  |  | S | 3.7 | | | | 1.9106 | -0.5 | 0.4494 | |
|  | 14 Days | D | -0.6 | | | | 0.3641 | -2.76059 | 1.64890 | |
|  |  | J | 0.348622 | | | | 0.5859 | 0.352621 | 0.5018 | |
|  |  | C | -1.19788 | | | | 1.591 | -0.5 | 0.594 | |
|  |  | S | 6.1 | | | | 4.2654 | 6.021229 | 3.193 | |
| IL-10 | 4 Days | D | -5.43131 | | | | 3.988 | 41.35814 | 12.435 | |
|  |  | J | 1.83796 | | | | 1.289 | 49.8 | 11.5624 | |
|  |  | C | -2.44528 | | | | 1.009 | -2.32593 | 1.208 | |
|  |  | S | 2.1386 | | | | 1.3987 | -1.62679 | 1.1093 | |
|  | 7 Days | D | -2.61531 | | | | 2.047 | 0.075967 | 1.4902 | |
|  |  | J | 2.227618 | | | | 2.734 | 79.18786 | 19.7643 | |
|  |  | C | -3.7 | | | | 2.456 | 1.385266 | 1.0754 | |
|  | 14 Days | S | 17.16073 | | | | 11.680 | 84.57059 | 25.3827 | |
|  |  | D | -3.28456 | | | | 1.94 | -0.68902 | 1.1745 | |
|  |  | J | -0.7061 | | | | 1.267 | 3.564868 | 2.603 | |
|  |  | C | -3.29083 | | | | 3.056 | -4.24468 | 3.8294 | |
|  |  | S | 1.5 | | | | 1.175 | 4.5 | 2.106 | |
| IL-6 | 4 days | D | -3.847 | | | | 5.43 | 30.5 | 14.26 | |
|  |  | J | -4.10007 | | | | 3.87 | 28.6 | 11.47 | |
|  |  | C | 0.256489 | | | | 1.017 | 1.109709 | 2.284 | |
|  |  | S | 3.6262 | | | | 3.837 | 10.39787 | | 8.3867 |
|  | 7 days | D | -3.58 | | | 1.584 | | 24.8 | | 8.75 |
|  |  | J | -4.382 | | | 2.890 | | 20.25 | | 11.17 |
|  |  | C | -0.5335 | | | 1.543 | | 1.020869 | | 1.286 |
|  |  | S | 6.5974 | | | 6.019 | | 5.434815 | | 6.308 |
|  | 14 days | D | -0.8.019 | | | 1.696 | | -0.7587 | | 1.346 |
|  |  | J | -2.5048 | | | 2.5684 | | -3.674 | | 3.372 |
|  |  | C | 2.308 | | | 1.943 | | 1.6484 | | 2.7978 |
|  |  | S | 1.3321 | | | 2.57 | | -0.6 | | 2.7964 |
|  |  | T | 38.08036 | | | 29.73 | | 131.6286 | | 50.8 |
| LITAF | 4 days | D | -2.86327 | | | 2.463 | | 1.124246 | | 2.4732 |
|  |  | J | -1.05129 | | | 1.82 | | -1.44076 | | 1.472 |
|  |  | C | 0.62097 | | | 2.94 | | 2.106742 | | 3.78974 |
|  |  | S | -8.42942 | | | 7.3837 | | -2.63338 | | 3.54876 |
|  | 7 days | D | -5.08934 | | | 6.8372 | | 1.067149 | | 3.473 |
|  |  | J | -1.92962 | | | 1.96 | | 89.40063 | | 33.4964 |
|  |  | C | 1.935189 | | | 1.74 | | 19.72784 | | 5.9532 |
|  |  | S | 8.258248 | | | 5.292 | | 4.003749 | | 3.5770 |
|  | 14 days | D | -9.2265 | | | 8.4692 | | 0.666662 | | 4.7687 |
|  |  | J | -1.12917 | | | 2.3932 | | 1.063621 | | 2.1047 |
|  |  | C | 3.993215 | | | 2.462 | | 2.30782 | | 3.674 |
|  |  | S | 2.470811 | | | 3.927 | | 6.389369 | | 8.1098 |
| IL-2 | 4 days | D | -0.37586 | | | 1.972 | | 120 | | 34.8665 |
|  |  | J | -12.553 | | | 6.854 | | 70.37067 | | 25.4309 |
|  |  | C | -0.78948 | | | 1.092 | | 1.369395 | | 1.397 |
|  |  | S | 8.536727 | | | 9.762 | | 4.440944 | | 5.1095 |
|  | 7 days | D | -1.3 | | | 3.76 | | 98.2 | | 29.58 |
|  |  | J | -0.5 | | | 1.38 | | 60 | | 20.17 |
|  |  | C | -11.0462 | | | 13.2892 | | 5.87093 | | 8.309 |
|  |  | S | 10.13583 | | | 12.8573 | | 4.445053 | | 7.209 |
|  | 14 days | D | -1.36 | | | 3.6978 | | 2.4624 | | 3.3826 |
|  |  | J | -3.443 | | | 3.3678 | | 2.4029 | | 3.011 |
|  |  | C | -2.12 | | | 2.127 | | -1.047 | | 2.650 |
|  |  | S | 8.857 | | | 9.570 | | 6.402 | | 8.1098 |
| IFN-γ | 4 days | D | -6.74702 | | | 10.3845 | | 2.575101 | | 3.3921 |
|  |  | J | 0.372963 | | | 1.14 | | 6.381194 | | 7.984 |
|  |  | C | -1.48165 | | | 2.37 | | 2.61624 | | 2.864 |
|  |  | S | 49.6931 | | | 33.16 | | 113.9046 | | 30.47 |
|  | 7 days | D | -5.02448 | | | 6.3718 | | -1.28992 | | 3.9840 |
|  |  | J | -1.59833 | | | 5.630 | | 364.3949 | | 111.53 |
|  |  | C | 4.500486 | | | 4.759 | | 19.69465 | | 12.5 |
|  |  | S | 200.35 | | 12.56 | | | 256.5833 | | 17.87 |
|  | 14 days | D | -2.78834 | | 2.3728 | | | 4.073818 | | 4.4826 |
|  |  | J | 4.655399 | | 6.372 | | | 136.75 | | 34.64 |
|  |  | C | 6.5142 | | 6.382 | | | 3.005438 | | 5.684 |
|  |  | S | 233.4243 | | 22.18 | | | 302.7369 | | 38.96 |
|  |  | T | 1.296873 | | 16.109 | | | 88.1 | | 35.36 |
| IL-12 | 4 days | D | -13.3818 | | 12.987 | | | -7.70966 | | 9.7948 |
|  |  | J | 0.721913 | | 2.278 | | | -4.53571 | | 5.029 |
|  |  | C | 23.27797 | | 20.0911 | | | 8.395438 | | 10.0291 |
|  |  | S | 4.201835 | | 4.384 | | | 1.904782 | | 3.092 |
|  | 7 days | D | -2.3 | | 2.109 | | | -3.71525 | | 3.178 |
|  |  | J | -1.57101 | | 4.290 | | | 153.1735 | | 43.48 |
|  |  | C | 2.005873 | | 4.309 | | | 155.9904 | | 53.96 |
|  |  | S | 6.4 | | 6.106 | | | 6.505095 | | 6.603 |
|  | 14 days | D | -8.9 | | 9.27 | | | -7.6 | | 7.48 |
|  |  | J | -13.048 | | 14.298 | | | -6.18053 | | 7.1056 |
|  |  | C | -4.95779 | | 3.292 | | | -13.4113 | | 12.3019 |
|  |  | S | 9.2 | | 6.378 | | | 2.122579 | | 4.105 |
| IL-4 | 4 days | D | -6.00387 | | 5.409 | | | 1.892827 | | 3.016 |
|  |  | J | -4.22938 | | 4.3029 | | | -12.2525 | | 13.7038 |
|  |  | C | 2.596198 | | 4.3987 | | | 44.47016 | | 40.184 |
|  |  | S | 0.619349 | | 1.3802 | | | -0.98 | | 1.3092 |
|  | 7 days | D | 16.23745 | | 14.38946 | | | 13.05057 | | 12.1074 |
|  |  | J | -3.35698 | | 3.3857 | | | 11.14572 | | 12.79 |
|  |  | C | 3.562047 | | 4.202 | | | 15.10727 | | 14.3578 |
|  |  | S | 1.44144 | | 1.10 | | | 3.143365 | | 3.305 |
|  | 14 days | D | 4.672056 | | 5.4892 | | | 7.029822 | | 7.372 |
|  |  | J | 9.383685 | | 10.07 | | | 22.90467 | | 20.1085 |
|  |  | C | 6.884002 | | 5.387 | | | 0.578265 | | 0.739 |
|  |  | S | 12.97 | | 13.018 | | | 14.87332 | | 17.403 |
| IL-5 | 4 days | D | -4.77669 | | 6.39872 | | | -2.93757 | | 4.2019 |
|  |  | J | -0.92259 | | 2.3028 | | | -6.71824 | | 5.6049 |
|  |  | C | -1.84647 | | 2.4039 | | | 1.366812 | | 2.3928 |
|  |  | S | 0.373992 | | 1.1093 | | | -2.17849 | | 2.334 |
|  | 7 days | D | -9.1 | | 8.302 | | | -4.00136 | | 5.392 |
|  |  | J | -1.81671 | | 2.1018 | | | 6.6 | | 8.40782 |
|  |  | C | -0.81584 | | 3.45763 | | | 5.8 | | 6.337 |
|  |  | S | 6.1 | | 7.284 | | | 2.300675 | | 3.4383 |
|  | 14 days | D | -5.6 | | 6.2187 | | | -2.3 | | 4.3937 |
|  |  | J | -5.85034 | | 6.1836 | | | -1.1 | | 2.382 |
|  |  | C | -2.92414 | | 4.04762 | | | -10.7542 | | 11.364 |
|  |  | S | 5.653145 | 6.174 | | | | -4.44422 | | 7.372 |
| IL-17 | 4 Days | D | -3.26918 | 4.480 | | | | -3.21529 | | 4.34018 |
|  |  | J | 0.770939 | 2.1037 | | | | -4.67285 | | 4.19746 |
|  |  | C | 1.3 | 2.56039 | | | | 0.207105 | | 1.483 |
|  |  | S | -4.69734 | 4.492 | | | | -2.56954 | | 2.476 |
|  | 7 Days | D | -6.98576 | 7.31912 | | | | -5.85902 | | 5.486 |
|  |  | J | -0.69294 | 1.1094 | | | | 0.981781 | | 1.296 |
|  |  | C | -1.85281 | 1.208 | | | | 4.677939 | | 5.298 |
|  |  | S | 1.179447 | 3.3022 | | | | -0.77918 | | 1.1095 |
|  | 14 Days | D | -5.19515 | 6.1096 | | | | -5.82887 | | 6.4028 |
|  |  | J | -2.15437 | 2.20187 | | | | -0.09983 | | 4.209 |
|  |  | C | -5.81859 | 3.3836 | | | | -3.56721 | | 6.1047 |
|  |  | S | 2.883695 | 3.0178 | | | | 2.033833 | | 3.0968 |
